# Staying Safe for the Long Haul: A Health Belief Model Analysis of COVID-19 Preventive Behaviors Through the Lens of Long COVID

**DOI:** 10.1177/10547738251360170

**Published:** 2025-08-05

**Authors:** Jeanine P.D. Guidry, Linnea I. Laestadius, Candace W. Burton, Paul B. Perrin, Carrie A. Miller, Melissa D. Pinto, Michael P. Stevens, Thomas Chelimsky, Raouf Gharbo, Gary S. Cuddeback, Kellie E. Carlyle

**Affiliations:** 1 Department of Communication and Cognition, Tilburg University, Tilburg, The Netherlands; 2 Joseph J. Zilber College of Public Health, University of Wisconsin – Milwaukee, Milwaukee, WI, USA; 3 School of Nursing, York University, Toronto, ON, Canada; 4 School of Data Science, Department of Psychology, University of Virginia, Charlottesville, VA, USA; 5 Department of Family Medicine and Population Health, Virginia Commonwealth University School of Medicine, Richmond, VA, USA; 6 Department of Nursing, Mayo Clinic, Phoenix, AZ, USA; 7 Department of Internal Medicine, Division of Infectious Diseases, West Virginia University School of Medicine, Morgantown, WV, USA; 8 Virginia Commonwealth University School of Social Work, Richmond, VA, USA; 9 Department of Social and Behavioral Sciences, Virginia Commonwealth University School of Public Health, Richmond, VA, USA

**Keywords:** long COVID, COVID-19, preventive behaviors, health belief model

## Abstract

Health problems associated with post-acute COVID-19, also known as “Long COVID,” range from mild to severe. The best defense against this potentially serious condition is to prevent COVID-19 infection and reinfection. The same preventive measures for COVID-19 may be used to help prevent the spread of Long COVID. This study used the Health Belief Model (HBM) to examine whether and how public understanding and awareness of Long COVID and its prevention shape the adoption of COVID-19 preventive behaviors. *N* = 605 English-speaking U.S.-based adults were recruited via Qualtrics. Predictors of intention to carry out COVID-19 preventive behaviors were investigated. Outcomes included behaviors relevant to preventing both acute and Long COVID. Across all models, except the one examining intent to get a vaccine booster, Black respondents were more likely than White respondents to express intent to carry out COVID-19 preventive behaviors. In addition, HBM constructs added significantly to the regression models. Susceptibility to Long COVID was significant for all behavioral outcomes (all *p*s < .05), self-efficacy for wearing a mask (*p* < .001), and self-efficacy for testing for COVID-19 after exposure and before a social event (*p*s < .001). In addition, perceived benefits for Long COVID prevention predicted intent of mask-wearing (*p* < .001), testing before a social event (*p* = .002), and getting a vaccine booster (*p* = .001). Perceived severity of Long COVID did not significantly predict adherence to preventive behaviors. U.S. adults are more likely to express intent to carry out COVID-19 preventive behaviors, such as masking and receiving booster vaccines, when they report feeling greater susceptibility to Long COVID as well as greater self-efficacy for engaging in these preventive behaviors. Public health messaging about Long COVID with incorporation of HBM constructs may be an effective means of increasing continued recommended COVID-19 preventive behaviors, which also hold co-benefits for prevention of infections, such as influenza and measles, as well as emerging viruses such as avian flu.

## Introduction

Post-acute sequelae of SARS-CoV-2 infection (PASC), better known as “Long COVID,” is an infection-associated chronic condition that occurs in individuals with prior SARS-CoV-2 (COVID-19) infection, where symptoms persist for at least 3 months (National Academies of Sciences & Medicine, 2024). Common symptoms include fatigue, cognitive dysfunction, shortness of breath, and various cardiac and neurological symptoms (Davis et al., 2021; Subramanian et al., 2022; van Kessel et al., 2022). As of September 2024, an estimated 17.9% of U.S. adults had experienced Long COVID (Centers for Disease Control and Prevention, 2024), which is also associated with functional impairment and a significantly reduced quality of life (Ballering et al., 2022; Centers for Disease Control and Prevention, 2024; Malik et al., 2022). Research also suggests that people of color may be affected by Long COVID more frequently and severely than White individuals (Ariza et al., 2024; Malesevic et al., 2023), exacerbating existing disparities in access to and utilization of health care.

As early as 2022, the U.S. Centers for Disease Control and Prevention (CDC) stated, “the best prevention of Long COVID and its related impacts remains to avoid infection and re-infection of COVID-19 by following basic interventions such as getting vaccinated and boosted, maintaining social distancing, wearing a mask, and handwashing” (Centers for Disease Control and Prevention, 2022). Since these behaviors prevent both COVID-19 infection and Long COVID, this study defined them collectively as “COVID-19 preventive behaviors.” These co-benefits suggest that Long COVID risk perception may be an underexplored avenue for increasing adoption of COVID-19 preventive behaviors to decrease instances of Long COVID as well as morbidity and mortality from acute COVID (Cooney, 2021). Although COVID-19 is no longer a global public health emergency, it remains a global health threat, with 330,000 new cases reported worldwide in November 2024 (World Health Organization, 2024), and new variants, such as the 2024 FLiRT strain and the 2025 NB 1.8.1 strain, reinforce the need for vigilance (Johns Hopkins University Bloomberg School of Public Health, 2024; Schnirring, 2025). Many COVID-19 preventive behaviors also prevent other infectious diseases like seasonal influenza and measles (Schmerling, 2025). Growing evidence links infectious diseases and chronic conditions (American Society for Microbiology, 2025; Choi et al., 2007), which underscores the need for targeted messaging to promote preventive behaviors. Psychosocial predictors of preventive behavior have been well-studied via the lens of health behavior theories, particularly the Health Belief Model (HBM), which posits that health behavior adoption depends on individuals’ perceived threat of illness and belief in the effectiveness of preventive actions (Rosenstock, 1974). Applied to Long COVID, key HBM constructs include perceived severity of and perceived susceptibility to Long COVID, perceived benefits of and barriers to preventive behaviors, self-efficacy to overcome barriers, and cues to carry out preventive behaviors. Since these behaviors also prevent acute COVID-19 morbidity and mortality, their adoption carries broader health benefits.

While many studies examine drivers of behavior to prevent acute COVID-19 (Guidry et al., 2021), few have considered behaviors specifically aimed at preventing Long COVID (Muratori Holanda et al., 2021), and little is known about factors shaping Long COVID-related risk perceptions (Muratori Holanda et al., 2021). The overarching aim of this study was to examine whether or not public understanding and awareness of Long COVID and its prevention influences the adoption of COVID-19 preventive behaviors. Given the very limited extant literature on Long COVID, we were not initially prepared to establish a predictive hypothesis about the relationship between these variables but posited a relationship between them based on our prior research on health prevention and help-seeking behaviors related to COVID-19 as well as other conditions (Guidry et al., 2021, 2022, 2024; Burton et al., 2022).

## Method

The Survey firm Qualtrics was used to recruit from existing research panels a survey sample of 605 English-speaking U.S. adults (ages 18–65), from April to May 2022. This sample was purposively identified with a quota of 52.1% reporting a COVID-19 diagnosis since March 2020, and 47.9% without a known COVID-19 infection. Additional quotas ensured a 50/50 split by sex assigned at birth and approximately one-third representation each of participants with White, Black, and Hispanic race/ethnicity. The study was approved by the Institutional Review Board at a large public research university in the Mid-Atlantic U.S.

### Measures

#### Demographics

Variables included age, sex assigned at birth, race/ethnicity, education, insurance status, and rurality.

#### COVID-19 Infection History

COVID-19 infection history was assessed by one question, “Have you been diagnosed with COVID-19 anytime since January 2020?” with answer options, “Yes, by a laboratory performed COVID PCR test,” “Yes, using a home rapid antigen test but NOT confirmed by a COVID PCR test,” and “No.” This variable was then dichotomized into a discrete “diagnosed/not diagnosed.”

#### Vaccination Status

COVID-19 vaccination status was measured using one item with the following four options: “I already received one or more doses of the COVID-19 vaccine,” “I am planning to get the COVID-19 vaccine as soon as possible,” “I am undecided about whether I will get the COVID-19 vaccine,” and “I will **not** get the COVID-19 vaccine.” These data were collapsed into a discrete “vaccinated/not vaccinated” variable, with “I already received one or more doses of the COVID-19 vaccine” and “I am planning to get the COVID-19 vaccine as soon as possible” classified as “vaccinated.” This was done in consideration of the relatively small and well-established intention-behavior gap and strong intention-behavior link for vaccinations in general and pandemic vaccinations in particular (Shiloh et al., 2022). By the same logic, the options, “I am undecided about whether I will get the COVID-19 vaccine” and “I will **not** get the COVID-19 vaccine,” were collapsed into a single “unvaccinated” category.

#### Health Belief Model

Participants responded to each of the items described below using a seven-point Likert scale that ranged from “*strongly disagree*” to “*strongly agree*” except for the question about ease of access to the vaccine in the self-efficacy domain, which used a seven-point Likert-type scale ranging from “*very difficult*” to “*very easy*.” All items were based on pandemic vaccine scales developed by Myers and Goodwin (2011) and on earlier scales developed by the authors (Guidry et al., 2021).

*Perceived severity* of Long COVID was assessed using four items (i.e., “Complications of Long COVID are serious,” “I could get very sick if I get Long COVID,” I am afraid of getting Long COVID,” and reverse coded: “Long COVID is easily treatable”). Cronbach’s alpha for items on the scale was .85.

*Perceived susceptibility* to Long COVID was measured using three items (e.g., “I am worried about the likelihood of getting Long COVID in the near future”). Cronbach’s alpha for items in the scale was .85.

*Perceived benefits to preventive measures* were measured using five items focused on the benefits of each of the preventive measures (COVID-19 vaccine, COVID-19 vaccine booster, mask wearing, and testing) related to Long COVID (e.g., “Vaccination will decrease my chance of getting Long COVID-19 or its complications”). Cronbach’s alpha for mask-wearing items was .91. Cronbach’s alpha for the items for testing after exposure was .81, and for testing before attending a social gathering was .87.

*Perceived barriers to preventive measures* were measured using five items related to mask wearing as prevention for Long COVID (e.g., “I don’t believe masks work to protect the person wearing them against Long COVID”); five items related to COVID-19 vaccine boosters as prevention for Long COVID (e.g., “I believe the COVID-19 vaccine boosters will have long-term harmful consequences”); and five items related to testing as prevention for Long COVID (e.g., “Taking a COVID-19 test is uncomfortable”). Cronbach’s alpha for mask wearing items was .90. Cronbach’s alpha for testing items was .83 (barriers for testing after exposure and testing before attending a social gathering combined due to similarity).

*Self-efficacy* related to preventive measures was measured by two items for each of the preventive behaviors. Question one was, “If I wanted to, I am confident that I could. . . (insert preventive behavior)” (responses ranging from “*strongly disagree*” to “*strongly agree*”), and question two was, “For me, the following actions will be. . .. (insert preventive behavior)” (responses ranging from “*Very difficult*” to “*Very easy*”). Cronbach’s alpha for mask-wearing items was .84. Cronbach’s alpha for testing was .86 (self-efficacy for testing after exposure and testing before attending a social gathering were combined due to similarity).

*Cues to action* for Long COVID was assessed by one item, “Has your healthcare provider talked to you about Long COVID and its prevention?” with response options “yes” and “no.”

### COVID-19 Preventive Behaviors

For those who indicated that they were already vaccinated with one or more doses of a COVID-19 vaccine, COVID-19 vaccine booster uptake was measured using one item with the following four options: “I already received one or more doses of the COVID-19 vaccine booster,” “I am planning to get the COVID-19 vaccine booster as soon as possible,” “I am undecided about whether I will get the COVID-19 vaccine booster,” and “I will not get the COVID-19 vaccine booster.” This measure was collapsed into a discrete “booster/no booster” variable, with “I already received one or more doses of the COVID-19 vaccine booster” and “I am planning to get the COVID-19 vaccine booster as soon as possible” classified as “booster” (while collapsing this variable may obscure interesting behavioral nuances, we again decided this considering the relatively small intention-behavior gap and strong intention-behavior link for vaccinations in general and pandemic vaccinations in particular; Shiloh et al., 2022) and “I am undecided about whether I will get the COVID-19 vaccine, booster” and “I will not get the COVID-19 vaccine booster” classified as “no booster.”

*Mask wearing, testing after COVID-19 exposure*, and *testing before attending a social gathering* were measured by asking respondents’ intent to carry out these behaviors in the coming 2 weeks, measured on a 5-item Likert scale ranging from “*very unlikely*” to “*very likely*.”

### Data Analytics Strategy

Following descriptive analyses performed using SPSS 28.0 (IBM), four linear hierarchical multiple regression analyses were used to explore how demographics, COVID-19 diagnosis status, COVID-19 vaccination status, and HBM constructs-related variables (related to prevention of as well as the perceived threat of Long COVID) predicted intent to engage in COVID-19 preventive behaviors (intentions to wear masks, get the vaccine booster, test after COVID-19 exposure, and test before attending a social gathering). There were no missing data in the dataset.

## Results

*N* = 605 survey responses were collected. Quotas successfully acquired a sample comprised of 48.6% males (*n* = 294) and 51.4% females (*n* = 311) and 34.7% (*n* = 210) White, 33.4% (*n* = 202) Black, and 31.9% (*n* = 193) Hispanic or Latinx participants, contributing to an extremely racially/ethnically diverse sample. The mean age of participants was 40.4 (*SD* = 13.7). Of the total sample, 85.6% (*n* = 518) reported having health insurance, while 14.4% (*n* = 87) did not; and 23.0% reported living in a rural area (*n* = 139), 38.8% in an urban area (*n* = 235), and 38.2% in a suburban area (*n* = 231), again reflecting good sample diversity. In addition, 66.0% of respondents reported they were vaccinated with the COVID-19 vaccine (*n* = 399), while 34.0% said they were not (*n* = 206), suggesting strong variability in this health behavior. Among those vaccinated, 63.2% reported having received a booster (*n* = 252) while 36.8% had not (*n* = 147). See complete sample characteristics in Table 1.

**Table 1.** Descriptives of the Sample (*N* = 605).

Characteristics	%	*n*
Education		
Bachelor’s degree or higher	23.8	144
Less than bachelor’s degree	76.2	461
Sex		
Male	48.6	294
Female	51.4	311
Race		
White	34.7	210
Black	33.4	202
Hispanic	31.9	193
Rurality		
Rural	23.0	139
Urban	38.8	235
Suburban	38.2	231
Health insurance		
Yes	85.6	518
No	14.4	87
COVID-19 vaccine status		
Vaccinated	66.0	399
Not vaccinated	34.0	206
Age	*M*	*SD*
Age, years	40.4	13.7

### Psychosocial Predictors of Preventive Behaviors

To investigate determinants of intention to carry out COVID-19 preventive behaviors, defined as behaviors relevant to preventing both acute and Long COVID, three hierarchical multiple linear regressions and one hierarchical logistic regression were carried out: the hierarchical linear regressions for predicting intent for mask-wearing in public settings, one for predicting intent for testing after exposure to COVID-19, one for predicting intent for testing before attending a social event, and the hierarchical logistical regression for predicting intent to obtain a COVID-19 booster vaccine if declared eligible (Tables 2–5).

**Table 2.** Hierarchical Multiple Regression Predicting Mask Wearing Intent.

Variable	β	*p*-value	Adjusted *R*^2^	β	*p*-value	Adjusted *R*^2^	β	*p*-value	Adjusted *R*^2^
Age	.161	<.001*	.107	.110	.012*	.126	.081	.034*	.386
Sex: female (Ref: male)	.032	.422		.031	.427		.053	.119	
Race: Black (Ref: White)	.367	<.001*		.353	<.001*		.239	<.001*	
Race: Hispanic (Ref: White)	.183	<.001*		.168	<.001*		.123	.003*	
Rurality: urban (Ref: rural)	.047	.355		.040	.430		.021	.617	
Rurality: suburban (Ref: rural)	−.041	.409		−.056	.261		−.056	.182	
Education: Bachelor’s (Ref: less than bachelor’s)	.009	.821		−.004	.915		−.054	.107	
Health insurance	−.066	.091		−.050	.197		−.013	.692	
COVID-19 diagnosis status: diagnosed (Ref: no COVID)				−.052	.211		−.007	.850	
COVID-19 vaccine status: vaccinated (Ref: not vaccinated)				.147	<.001*		.007	.838	
HBM: Long COVID severity							−.026	.527	
HBM: Long COVID susceptibility							.174	<.001*	
HBM: barriers of mask wearing							−.192	<.001*	
HBM: benefits of mask wearing							.223	<.001*	
HBM: self-efficacy mask wearing							.274	<.001*	
HBM: Long COVID cue to action							.054	.127	

*Note.* *p < .05.

**Table 3.** Hierarchical Multiple Regression Predicting Testing After Exposure Intent.

Variable	β	*p*-value	Adjusted *R*^2^	β	*p*-value	Adjusted *R*^2^	β	*p*-value	Adjusted *R*^2^
Age	.062	.149	.027	.020	.659	.036	−.049	.257	.229
Sex: female (Ref: Male)	.025	.547		.021	.617		−.019	.620	
Race: Black (Ref: White)	.179	<.001*		.169	<.001*		.099	.025*	
Race: Hispanic (Ref: White)	.078	.119		.072	.153		.035	.439	
Rurality: urban (Ref: rural)	.038	.477		.032	.549		.009	.851	
Rurality: suburban (Ref: rural)	.005	.922		−.006	.911		.001	.975	
Education: Bachelor’s (Ref: less than bachelor’s)	.022	.597		.016	.706		−.016	.667	
Health insurance	−.101	.013*		−.091	.026*		−056	.128	
COVID-19 diagnosis status: diagnosed (Ref: no COVID)				−.064	.146		−.058	.153	
COVID-19 vaccine status: vaccinated (Ref: Not VACCINATED)				.099	.020*		−.044	.274	
HBM: Long COVID severity							.161	<.001*	
HBM: Long COVID susceptibility							.117	.008*	
HBM: barriers testing after exposure							−.109	.014*	
HBM: benefits testing after exposure							.090	.041*	
HBM: self-efficacy testing							.236	<.001*	
HBM: Long COVID cue to action							−.018	.650	

*Note.* *p < .05.

**Table 4.** Hierarchical Multiple Regression Predicting Testing Before Social Event Intent.

Variable	β	*p*-value	Adjusted *R*^2^	β	*p*-value	Adjusted *R*^2^	β	*p*-value	Adjusted *R*^2^
Age	.016	.709	.065	−.004	.932	.074	−.010	.817	.253
Sex: female (Ref: male)	−.007	.868		.002	.965		−.006	.868	
Race: Black (Ref: White)	.257	<.001*		.248	<.001*		.176	<.001*	
Race: Hispanic (Ref: White)	.139	.005*		.116	.020*		.089	.049*	
Rurality: urban (Ref: rural)	.048	.352		.046	.380		.023	.617	
Rurality: suburban (Ref: Rural)	−.081	.112		−.090	.080		−.067	.146	
Education: Bachelor’s (Ref: less than bachelor’s)	.062	.127		.046	.262		.009	.800	
Health insurance	−.069	.086		−.056	.164		−.010	.785	
COVID-19 diagnosis status: diagnosed (Ref: no COVID)				.026	.538		−.018	.652	
COVID-19 vaccine status: vaccinated (Ref: not vaccinated)				.111	.008*		.003	.940	
HBM: Long COVID severity							.023	.620	
HBM: Long COVID susceptibility							.207	<.001*	
HBM: barriers testing before event							−.068	.119	
HBM: benefits testing before event							.123	.005*	
HBM: self-efficacy testing							.187	<.001*	
HBM: Long COVID cue to action							−.138	<.001*	

*Note.* *p < .05.

**Table 5.** Hierarchical Multiple Regression Predicting COVID-19 Vaccine Booster Uptake Intent.

Variable	β	*p*-value	Adjusted *R*^2^	β	*p*-value	Adjusted *R*^2^	β	*p*-value	Adjusted *R*^2^
Age	.015	.068*	.044	.087	.126	.044	.004	.942	.195
Sex: female (Ref: Male)	.004	.936		−.006	.901		.016	.723	
Race: Black (Ref: White)	.044	.465		.037	.537		.043	.408	
Race: Hispanic (Ref: White)	−.015	.692		−.017	.795		−.041	.457	
Rurality: urban (Ref: rural)	.026	.697		.026	.707		.024	.678	
Rurality: suburban (Ref: rural)	.024	.726		.021	.309		.031	.590	
Education: Bachelor’s (Ref: less than bachelor’s)	.074	.145		.081	.110		.031	.474	
Health insurance	−.009	.865		−.012	.806		.012	.778	
COVID-19 diagnosis status: diagnosed (Ref: no COVID)				−.075	.178		.003	.947	
HBM: Long COVID severity							.012	.817	
HBM: Long COVID susceptibility							.134	.008*	
HBM: barriers booster uptake							−.282	<.001*	
HBM: benefits booster uptake							.199	<.001*	
HBM: self-efficacy booster uptake							.297	<.001*	
HBM: Long COVID cue to action							.086	.064	

*Note.* *p < .05.

#### Mask Wearing

The *R*^2^ for Step 1, predicting intent to wear a mask in public settings, which included demographic variables, was .119, with the variables explaining 11.9% of the variance in the intention to wear a mask, *F*(8,596) = 10.02, *p* < .001. The addition of COVID-19 diagnosis status and vaccination status in Step 2 was significant, though a somewhat negligible addition, *R*^2^ = .14, *F*(10,594) = 9.71, *p* < .001. The addition of HBM variables in Step 3 was also significant and contributed to substantial variance explained, *R*^2^ = .40, *F*(16,588) = 24.77, *p* < .001, for a large-sized effect.

#### Testing After Exposure to COVID-19

The *R*^2^ for Step 1, predicting intent to test after exposure to COVID-19, which included demographic variables, was .040, with the variables explaining 4.0% of the variance in the intention to test after exposure to COVID-19, *F*(8,596) = 3.08, *p* = .002. The addition of COVID-19 diagnosis status and vaccination status in Step 2 was significant, though again a fairly negligible addition, *R*^2^ = .052, *F*(10,594) = 3.26, *p* < .001. The addition of HBM variables in Step 3 was also significant and contributed to a substantial increase in variance explained, *R*^2^ = .242, *F*(16,588) = 11.73, *p* < .001, for an overall medium-sized effect.

#### Testing Before Attending an Event

The *R*^2^ for Step 1, testing before attending a social event, which included demographic variables, was .078, with the variables explaining 7.8% of the variance in the intention to test before attending a social event, *F*(8,596) = 6.29, *p* < .001. The addition of COVID-19 diagnosis status and vaccination status in Step 2 was significant, though a minor contributor, *R*^2^ = .09, *F*(10,594) = 5.83, *p* < .001. The addition of HBM variables in Step 3 was also significant and again substantial, *R*^2^ = .27, *F*(16,588) = 13.83, *p* < .001, an overall large-sized effect.

#### COVID-19 Vaccine Booster

The *R*^2^ for Step 1, predicting intent to get a COVID-19 vaccine booster when eligible among those who had already received a vaccine, which included demographic variables, was .044, with the variables explaining 4.4% of the variance in the intention to get a COVID-19 vaccine booster when eligible, *F*(8,596) = 12.992, *p* = .112. The addition of COVID-19 diagnosis status in Step 2 was not significant, *R*^2^ = .044, *F*(9,595) = 13.185, *p* = .661. The addition of HBM variables in Step 3, however, was significant, *R*^2^ = .195, *F*(16,588) = 61.471, *p* < .001, an overall large-sized effect.

Across all models except for intent to get the booster vaccine, Black respondents were more likely than White respondents to express intent to carry out COVID-19 preventive behaviors. In addition, compared to White respondents, Hispanic respondents were more likely to express intent to wear a mask in public settings as well as test after exposure to COVID-19. Other demographic variables – age, sex, education, and rurality – did not predict intent to carry out these four preventive behaviors in the final models.

For HBM constructs, in all four models, a higher score for perceived susceptibility to Long COVID predicted higher intent to carry out each behavior. Self-efficacy to carry out these preventive behaviors was a significant predictor of intent for all four behaviors, and perceived benefits of each behavior predicted intent to carry out the corresponding behaviors. Finally, high perceived barriers to mask wearing and to getting the booster vaccine were significant predictors of lower intent to carry out these two behaviors. Perceived severity of Long COVID was only a significant predictor for intent to test after exposure, and cues to action was only a significant predictor for intent to test before attending a social event.

## Discussion

This study examined how public understanding and awareness of Long COVID and its prevention influences engagement in COVID-19 preventive behaviors. In an environment where individuals must now assess their own COVID-19 risk (Fisher, 2022), formal public health mandates have been lifted, and new COVID-19 variants appear regularly (Johns Hopkins University Bloomberg School of Public Health, 2024). Understanding the drivers of voluntary compliance with infection mitigation guidelines is critical. Findings indicate that U.S. adults are more likely to express intent to carry out COVID-19 preventive behaviors, such as masking and booster vaccinations, when they perceive susceptibility to Long COVID and report greater self-efficacy to engage in these behaviors. Increased awareness of the role of these behaviors in preventing Long COVID also increased behavioral intent. Notably, Long COVID-related perceptions significantly influenced intent for masking and testing even when controlling for COVID-19 vaccination status. This suggests people remain receptive to health communication about Long COVID prevention post-vaccination. This provides an encouraging sign for public health and medical practitioners, as vaccines reduce but do not eliminate the risk of Long COVID entirely (Notarte et al., 2022).

Our findings are notable because they suggest continued public willingness to engage in preventive behaviors in April 2022, when few mask mandates remained (Cancryn & Owermohle, 2022). Further, that same year, the CDC updated its masking guidelines, no longer recommending masking for the general population (Centers for Disease Control and Prevention, 2025a). Paired with this change, the CDC noted that “At all levels, people can wear a mask based on personal preference, informed by personal level of risk” (Centers for Disease Control and Prevention, 2025b). Nevertheless, as of March 2025, the CDC still recommended staying up to date with COVID boosters and practicing good hygiene, with mask wearing now framed as an “additional prevention strategy that you can choose to further protect yourself and others” (Centers for Disease Control and Prevention, 2025b). As of May 2025, CDC guidelines appear to be further shifting against recommending universal preventive behaviors (Centers for Disease Control and Prevention, 2025b). This study’s findings suggest that public education to create more accurate perceptions of the potential impacts of Long COVID may encourage adoption of evidence-based COVID-19 preventive behaviors even in the absence of government mandates and shifting recommendations.

To date, however, Long COVID has been largely overlooked in state-level COVID-19 messaging (Laestadius et al., 2022), and public concern about its connection to preventive behaviors remains limited (YouGov, 2022). The reasons for this gap warrant further exploration. Campaigns emphasizing only the risks of acute COVID-19 morbidity and mortality rather than post-acute consequences may contribute to lower adoption of preventive behaviors such as masking and testing, especially in the light of lower societal perceived threat of COVID-19. Given the emergence of new COVID-19 variants such as NB1.8.1 (Schnirring, 2025) and the now-endemic nature of COVID-19, it is imperative that health researchers and providers advocate for measures that reduce both acute COVID-19 morbidity and its associated incidence of Long COVID.

No significant racial or ethnic differences were observed in booster uptake, aligning with newer data suggesting that vaccine hesitancy has declined more rapidly among Black adults than White adults (Padamsee et al., 2022). However, Black and Hispanic/Latinx respondents were more likely than their non-Hispanic White counterparts to mask and test before events. This is consistent with prior research and may be attributable to stronger prosocial beliefs and concern for communal wellbeing among these populations (Choi et al., 2022; Orom et al., 2021). For example, prior research on COVID preventive behaviors indicates that Black and Latinx adults placed a greater importance on protecting others from COVID than White adults (Orom et al. 2021). However, it should be noted that behaviors such as mask wearing occur within a broader social context that may create social and personal risks. For example, early in the pandemic, Black adults reported higher levels of concern about racial stereotyping by both the public and police in relation to mask wearing as compared to White adults (Kahn & Money, 2022). These concerns correlated with lower adoption of masking behaviors even while their overall rate of masking continued to exceed that of White adults. Future work is needed to more explicitly understand how barriers and enabling factors to Long COVID preventive behaviors may differ by race and ethnicity.

In addition, in the United States, the impact of the COVID pandemic has been shown to have greater and more far-reaching implications among Black and Latinx communities with regard to mortality, overall life expectancy, and severity of illness (Andrasfay & Goldman, 2022; Berkowitz et al., 2021). These communities are also historically more likely to experience poverty, under- or unemployment, and reduced access to health care – all considered social determinants of health according to the U.S. Department of Health and Human Services (Burton et al., 2020; Voss et al., 2023). While these influences often have negative effects on individual capacity for health maintenance, some studies suggest that community influence in marginalized populations can prompt greater engagement with preventive health activities such as vaccination (Padamsee et al., 2022). These factors may also contribute to heightened risk perception and resulting in other preventive behaviors. For example, prior research indicates that Latinx adults were more likely to self-report heightened COVID exposure risk via their workplace and avoid in-person work relative to White adults (Orom et al., 2021). This may further contextualize our findings.

More broadly, findings indicate that public health messaging about Long COVID and the current options for preventing Long COVID may be an effective means of increasing COVID-19 preventive behaviors that are effective against both acute COVID-19 and Long COVID. This also holds implications for other infectious diseases that are known to pose a risk of chronic disease, but that have been primarily framed as an acute condition, such as Epstein-Barr virus and Varicella-zoster virus. While prior research suggests that acute infectious diseases are often seen by the public as more concerning than chronic diseases (De Zwart et al., 2009), the findings here suggest that fully informing the public about both acute and post-acute risks from infectious diseases may increase willingness to engage in preventive behaviors.

### Strengths, Limitations, and Future Directions

This study is among the first to examine Long COVID perception as a predictor of adherence to COVID-19 preventive behaviors. A key strength is the diverse sample by both sex and race/ethnicity. However, there are some limitations that inform directions for future research. First, the cross-sectional design limits determination of directionality of associations (Spector, 2019). This constrains our ability to draw firm conclusions about the causal order of observed relationships. While Long COVID perceptions or HBM constructs may influence preventive behaviors, the reverse may also be true. Future longitudinal research using cross-lagged panel designs could help infer causality among the variables under scrutiny. Second, potentially relevant variables such as COVID-19 severity among those who had tested positive and COVID-19 on friends or family were not measured. Future research would benefit from assessing the influence of these variables on the associations identified in the current study. Third, the panel sample, though diverse, is not nationally representative. In addition, while quota sampling helps ensure representation of key subgroups, it limits the generalizability of the study (Bornstein et al., 2013). Finally, while the intention-behavior gap is relatively small and the link between intention and behavior strong for pandemic vaccinations, TPB’s intention-behavior gap may still be a limitation (Conner & Norman, 2022), and future studies should consider using actual behavior as a study outcome. Overall, future research should assess the effectiveness of messaging campaigns emphasizing acute risks of COVID-19 infection, post-acute risks, or both.

## Conclusion

The results of this study hold promise both for increasing COVID-19 preventive behaviors through better messaging about Long COVID, and for messaging to prevent infection-associated chronic diseases more broadly. Notably, our findings suggest that Long COVID concern influences preventive behaviors among both the vaccinated and unvaccinated. One likely pathway to increasing adoption of COVID-19 preventive behaviors is increasing awareness and understanding of Long COVID. Emphasizing both the efficacy of vaccine boosters for preventing morbidity and mortality from acute COVID-19 *and* the risks of Long COVID may therefore be an effective strategy for encouraging adherence to recommended preventive behaviors. This may, in turn, prevent both the most serious consequences of acute COVID-19 infection, as well as mitigate the devastating trajectories of Long COVID. In addition to preventing COVID-19 and Long COVID, preventive behaviors such as social distancing and masking also hold co-benefits for prevention of infections such as influenza and measles, as well as emerging viruses such as avian flu.
